# A cell-extrinsic ligand acquired by activated T cells in lymph node can bridge L-selectin and P-selectin

**DOI:** 10.1371/journal.pone.0205685

**Published:** 2018-10-31

**Authors:** Douglas A. Carlow, Michelle C. Tra, Hermann J. Ziltener

**Affiliations:** 1 The Biomedical Research Centre, Faculty of Medicine, University of British Columbia, Vancouver, British Columbia, Canada; 2 Department of Pathology, Faculty of Medicine, University of British Columbia, Vancouver, British Columbia, Canada; Ludwig-Maximilians-Universitat Munchen, GERMANY

## Abstract

P-selectin expressed on activated endothelia and platelets supports recruitment of leukocytes expressing P-selectin ligand to sites of inflammation. While monitoring P-selectin ligand expression on activated CD8^+^ T cells in murine adoptive transfer models, we observed two distinct ligands on responding donor cells, the canonical cell-intrinsic P-selectin ligand PSGL-1 and a second undocumented P-selectin ligand we provisionally named PSL2. PSL2 is unusual among selectin ligands in that it is cell-extrinsic, loaded onto L-selectin expressed by activated T cells but not L-selectin on resting naïve CD8^+^ T cells. PSL2 display is highest on activated T cells responding in peripheral lymph nodes and low on T cells responding in spleen suggesting that the original source of PSL2 is high endothelial venules, cells known to produce L-selectin ligands. PSL2 is a ligand for both P-selectin and L-selectin and can physically bridge the two selectins. The L-selectin/PSL2 complex can mediate P-selectin-dependent adherence of activated T cells to immobilized P-selectin or to activated platelets, either independently or cooperatively with PSGL-1. PSL2’s capacity to bridge between L-selectin on activated T cells and P-selectin reveals an undocumented and unanticipated activity of cell-extrinsic selectin ligands in mediating selectin-selectin connectivity. The timing and circumstances of PSL2 detection on T cells, together with its capacity to support adherence to P-selectin-bearing substrates, are consistent with P-selectin engagement of both PSGL1 and the L-selectin/PSL2 complex during T cell recruitment. Engagement of PSGL-1 and L-selectin/PSL2 would likely deliver distinct signals known to be relevant in this process.

## Introduction

Leukocyte tethering to endothelium is the initial step in movement of leukocytes from blood into tissue—a fundamental process in lymphoid homeostasis, the inflammatory response, and immunological defense. These tethering interactions begin with low affinity contacts between leukocytes and activated vascular endothelia through binding of selectins to their ligands on opposing cell surfaces. Identification of all physiologically relevant selectin ligands is needed to complete the understanding of selectin function in the aforementioned fundamental processes.

P-selectin and E-selectin [1] are expressed on activated endothelium and tether to ligands expressed on leukocytes to support their recruitment during inflammation [2–4]. P-selectin is also expressed at high density on activated platelets and cyclically on thymic endothelium[5]. All selectins recognize ligands modified with sialyl-Lewis X (sLex) tetrasaccharides but P-selectin, E-selectin and L-selectin each engage largely distinct ligand sets determined by additional modifications of the sLex glycan and properties of the scaffold or peptide backbone. P-selectin is generally thought to have a single, broadly utilized and physiologically active ligand, Platelet Selectin Glycoprotein Ligand 1 (PSGL1). However, P-selectin recognition of PSGL1 also requires that sLex be presented on a branched O-glycan together with sulfated tyrosine residues adjacent to the O-glycan attachment site. This branched O-glycan on PSGL1 is generated in the golgi by the enzyme Core 2 β1,6 glucosaminyl N-acetyl Transferase 1 (C2GnT1). Such ‘decorated’ PSGL1 P-selectin ligand is present constitutively on neutrophils but induced on T lymphocytes only after their antigen-driven activation in secondary lymphoid organs, an event that corresponds with induction of the C2GnT1 enzyme. Thus, induction of PSGL1 P-selectin ligand expression constitutes part of the *cell-intrinsic* response by lymphocytes to support recruitment via P-selectin on vasculature of inflamed tissue.

While studying formation of PSGL1 P-selectin ligand on primary in vivo activated CD8^+^ T cells (here referred to as ‘activated T-cells’) we detected a second PSGL1-independent P-selectin ligand and provisionally named it P-selectin-Ligand-2 (PSL2). Like decorated PSGL1, PSL2 was reliably detected on CD8^+^ T cells after activation in peripheral lymph nodes. The contemporaneous expression of the two selectin ligands, PSGL1 and PSL2, on activated T cells positions these two ligands to cooperate during encounter with P-selectin. However, in contrast to PSGL1 and the vast majority of other selectin ligands, PSL2 was found to be *cell-extrinsic*; PSL2 is acquired by T cells activated in lymph nodes by docking onto L-selectin. We describe how PSL2 was discovered and characterized.

## Materials and methods

### Mice

Male and female mice aged 8–12 weeks were used for analyses. C57BL/6 (B6) mice were bred from founders obtained originally from Jackson Laboratory, Bar Harbour, Maine. *PSGL-1*^*null*^ (B6.Cg-Selplgtm1Fur/J stock #004201) and *P-selectin*^*null*^ mice on the B6 background, and *Thy1*.*1* mice were also obtained from Jackson Laboratory. *C2GnT1*^*null*^ mice[6] backcrossed with B6 mice beyond F7 were provided by Dr. Jamey Marth, University of California at Santa Barbara. T cell receptor transgenic *OT1*[7] and *HY*[8] mice were backcrossed beyond F8 on B6 background. *L-selectin*^*null*^ mice were provided by Dr. Steve Rosen (University of California at San Francisco). Mice were bred at the specific pathogen-free animal facility at the Biomedical Research Centre, University of British Columbia. Procedures employed in this study were approved by the Animal Care Committee at the University of British Columbia.

### Media and salt solutions

Routine media was designated I_10_ and included Iscove’s Modified Dulbeco’s Media (IMDM; Gibco Life Technologies #12440–046) supplemented with 10% fetal bovine serum (various suppliers), 100 U/ml penicillin, 100 U/ml streptomycin (Stem Cell Technologies), 2 mM glutamine (Sigma-Aldrich), and 5x10^-5^ M beta-mercaptoethanol (BioRad # 1610710). Dulbecco’s Modified Eagle Media (DMEM) Gibco #11965–084 supplemented with 20mM HEPES (Sigma Aldrich #H4034 pH 7.2) and 2 mg/ml bovine serum albumin (BSA) was used when staining with biotinylated antibodies. Calcium-free and magnesium-free phosphate buffered saline pH 7.4 (PBS) was prepared in-house. Hanks balanced salt solutions with Mg^++^ and Ca^++^ referred to here as ‘H+’ (Gibco Life Technologies #14025–092) or without Mg^++^ and Ca^++^ referred to here as ‘H-‘ (Gibco Life Technologies #14170–112) were supplemented as indicated with bovine serum albumin (BSA; Sigma #A7906), EDTA pH 7.4, (BioRad #161–0728), CaCl_2_ (EMD #10035-04-8), MgCl_2_ (Fisher #M33-500), or MnCl_2_ (J. T. Baker Inc. #2540–04). H- B2 (H- with 2mg/ml BSA), H- B5 E2 (H- with 5 mg/ml BSA + 2mM EDTA), H- B2 E2 (H- with 2 mg/ml BSA + 2mM EDTA), H- B5 H10 Ca0.5 (H- with 5 mg/ml BSA + 10mM HEPES + 0.5 mM CaCl_2_).

### In-vitro T cell stimulations

Pooled lymph node and spleen cells were activated with 5μg/ml Concanavalin A for two days and then sub-cultured with fresh media and interleukin 2 for two more days. Dendritic cells from B6 or PSGL-1^*null*^ mice used for in vitro stimulations were prepared by differential adherence to plastic as previously described[9] except that spleen cell suspensions were not subjected to red cell lysis or filtered prior to plating. Dendritic cell-enriched suspensions were harvested after overnight detachment from petri plastic and either pulsed for 30 minutes at 37°C with 5 mg/ml ovalbumin in I_10_ or incubated for 60 minutes on ice with 10 μg/ml HY peptide KCSRNRQYL[10]. Dendritic cells were then washed, counted, and cultured at 2x10^4^ cells/well together with either 3-10x10^4^ CD4-depleted HY thymocytes or 2x10^4^ OT1 lymph node cells. After three days, cultures were harvested and stained.

### Antibodies, selectin chimera staining, tracking dyes and flow cytometry

#### Antibodies

CD62L clone Mel-14 biotin (in house); anti-L-selectin ligand Meca79 (Biolegend #120804) and IgM isotype control (Biolegend #400803); CD44-FITC clone IM7.8.1 (in-house); Ly6C-APC (eBioscience #17–5932); CD8 clone 53–6.7 conjugates (APC, eBioscience #17–0081); Alexafluor-649 (in house); biotin (in-house); APC-EFluor-780, eBioscience 47-0081-82; PE, eBioscience #12-0081-85; FITC (in-house); Thy1.2 (TIB 107) Fab fragment biotin (in-house); CD4-biotin clone GK1.5 (in-house);CD19-biotin clone 1D3 (in-house); CD41-APC (Biolegend #133914); Streptavidin conjugates (APC, eBiosciences #17-4317-82; PE-Cy7, eBioscience #25-4317-82; PE, BD-Pharmingen #554061). Propidium iodide (PI) was used at 200 ng/ml to label dead cells.

#### Selectin chimeras

Prior to selectin-hIgG chimera staining, ex-vivo lymphocytes were usually depleted of sIg^+^ cells. Lymphocytes were stained for 20–30 minutes on ice with hIgG_1_ chimeras of mouse P-selectin (BD Pharmingen 555294), mouse E-selectin (R&D systems 575-ES), or mouse L-selectin (R&D systems 576-LS) at 5 μg/ml in I_10_ media ± 10mM EDTA. Cells were washed twice and stained with R-phycoerythrin-conjugated goat-anti-human IgG Fc (Jackson ImmunoResearch #109-115-098), washed, and usually co-stained with fluorochrome conjugated anti-CD8.

#### Tracking dyes

Two tracking dyes were used follow generation of donor-derived activated T cells 3 days after adoptive transfer. Prior to transfer ex-vivo donor cells to be labeled with tracking dyes were pelleted from I_10_ media and re-suspended in 37°C H+ containing either 2μM CFDA-SE (CFSE, InVitrogen C1157) or 5μM Cell Trace Violet (CTV, InVitrogen C34557), incubated at room temperature for 5 minutes, pelleted, washed once in H+, re-suspended in H+, and injected into recipients.

#### Flow cytometry

Flow analysis with FacsCalibur and LSRII (Becton Dickenson). FCS file analysis was conducted with FlowJo software. Dead cells, debris, and aggregates were excluded from analysis by gating of forward scatter and propidium iodide negative signals. Doublet discrimination was applied to exclude doublets in data collected with LSRII.

### Adoptive transfers, activated T cells harvest, and counting

For OT1-based adoptive transfer responses, donor cell suspensions were prepared from pooled peripheral and mesenteric lymph nodes, depleted of surface Ig^+^ cells with Dynabeads sheep-anti-mIgG (Invitrogen #11031), and labeled with tracking dyes prior to intravenous transfer of 5x10^6^ cells in H+ with 1mg ova antigen into recipients. For HY-based male antigen specific responses, CD4-depleted donor HY thymus tissue was used. Donor thymus cell suspensions were prepared in I_10_ and depleted of CD4^+^ cells using anti-CD4 GK1.5 pre-loaded onto Dynabeads Sheep-anti-Rat-IgG (Invitrogen #11035) and labeled with tracking dyes prior to intravenous injection of 5x10^6^ donor cells per recipient. Three days later, lymphoid tissues were harvested, cell suspensions prepared in I_10_, and depleted of sIg^+^ cells (as above) to enrich for activated T cells prior to selectin staining. Unless otherwise indicated, in vivo activated T cells used in this study were obtained from peripheral lymph nodes excluding mesenteric nodes. To assess activated T cells responses in tracheo-bronchial lymph node, OT1-based *L-selectin*^*-/-*^ and *L-selectin*^*+/+*^ donor cells were labeled with distinct tracking dyes, and co-injected intravenously into recipients. At the time of donor cell iv injections, recipient mice also received an intraperitoneal injection of 30 μl of stock 1 μm polystyrene bead suspension (PolySciences #17154) that had been pre-coated for 20 minutes with 10mg/ml ovalbumin in PBS and washed in PBS. Where indicated, donor activated T cells cell yields were monitored at day 3 using a flow cytometry-based counting assay using 10 μm CML latex beads (InVitrogen C37259) as previously described [11].

### EDTA treatments

Two distinct EDTA treatments were conducted. Inclusion of 10mM EDTA during selectin chimera staining was used as a control for selectin binding via its lectin domain. EDTA pre-wash was also performed to ‘strip’ PSL2 from activated T cells. Cells at ≤2x10^7^/ml in I_10_ were stripped of PSL2 by adding EDTA to 10mM, rested on ice for 5 minutes, vortexed gently for 5 seconds, pelleted, re-suspended in H- B5 E2, rested another 5 minutes on ice, re-vortexed gently for 5 seconds, pelleted, re-suspended in I_10_, and filtered through a 70μm nylon screen.

### PSL2 rebinding assay

CD8^+^ *HY-C2GnT1*^*null*^ donor activated T cells recovered from lymph nodes of male *PSGL1*^*null*^ *Thy1*.*1* recipients on day 3 were enriched by positive selection with biotinylated anti-Thy1.2 Fab and anti-PSGL1 4RA10 Fab antibodies using an EasySep Biotin Selection Kit (Stem Cell Technologies #18556). Cells were briefly washed 1x with 1 ml of H- B2, and then re-suspended at 10^7^/ml in H- B2 E2 for 15 minutes on ice with brief gentle vortexing at 5 minute intervals. Cells were then pelleted out of suspension and separated from the supernatant by centrifugation. The supernatant was then spun at 16,000g for 1 minute to clear residual debris and supplemented with CaCl_2_ to 3mM Ca^++^ (yielding 1mM free Ca^++^). The aliquots of stripped cells were re-suspended in 75μl I_10_ media, divided into three equal aliquots, and incubated for 60’ on ice with (i) Ca^++^ replete supernatant, (ii) an equal volume of I_10_ media, or (iii) an equal volume of mock supernate generated as Ca^++^ replete supernatant but never exposed to cells. Cells where then washed in I_10_ and stained with P-selectin-hIgG.

### In vitro PSL2 transfer

*OT1-C2GnT1*^*null*^ donor cells were labeled with CFSE or CTV tracking dyes, adoptively transferred into either *PSGL1*^*null*^ or *C2GnT1*^*null*^ recipients, and recipient lymph nodes harvested at day 3. Cell suspensions contained CFSE-diluted PSL2^+^ activated T cells or CTV-diluted PSL2^negative^ activated T cells respectively. The latter cells were enriched for donor activated T cells by depleting whole lymph node cells with Dynabeads Sheep-anti-Rat-IgG (above) pre-loaded with rat antibodies specific for CD4 (GK1.5) and CD19 (ID3). PSL2 transfer to donor-derived PSL2^negative^ enriched cells was observed after mixing and 4x repeated pelleting/re-suspension of 0.5x10^6^ of these with 10^6^ whole lymph node suspensions from the *OT1-C2GnT1*^*null*^ → *PSGL1*^*null*^ adoptive transfer.

### Platelet binding assay

#### Platelet preparation

Platelets were prepared with all solutions at room temperature. Blood from B6 and *P-selectin*^*null*^ donors was harvested by heart puncture after Avertin anesthesia as follows. Approximately 1 ml of blood was drawn into a 3 ml syringe containing 150μl acid citrate dextrose solution (ACD; 22g/L trisodium citrate 2H_2_O, 8 g/L Citric acid H_2_O; 24.5 g/L dextrose), mixed by inversion, supplemented with 200μl PBS containing 7mM EDTA, contents mixed and transferred into a 1.2 ml polypropylene cluster tube (#4401, Corning Incorporated, New York). Tubes were spun at 340g for 4 minutes and decelerated without brake. Platelets concentrated in the middle, cloudy, RBC-free layer (platelet rich plasma) were harvested in < 200μl with a P200 Gilson pipette and added to 2 ml of PBS containing 7mM EDTA in a 5 ml polystyrene Falcon tube (Corning #352054). Platelets were re-spun at 1800g for 5 minutes with deceleration at lowest brake setting. Supernatant was discarded and platelets re-suspended in 500μl H- B5 E2, labeled with 3μl of anti-CD41-APC for 5 minutes, added 2 ml H- B2, and re-pelleted at 1800g for 5 minutes, again at lowest brake setting. The supernatant was discarded and platelets re-suspended in 2ml PBS with 0.1mM CaCl_2_. Bovine thrombin (Sigma #T6634) was added to 1 Unit/ml from a thawed 100 Unit/ml stock solution and the suspension incubated at 37°C for 4 minutes. Two millilitres of room temperature PBS containing 2% paraformaldehyde (Electron Microscopy Sciences #15710) was added, mixed, and incubated for 10 minutes at room temperature, transferred to a BSA-blocked 5ml tube containing 200 mg/ml BSA in PBS to yield a final BSA concentration of 10 mg/ml, and pelleted for 8 minutes at 1800g. Fixed and labeled platelets were then re-suspended in H- B5 H10 Ca0.5, counted, pelleted, and re-suspended to 5x10^8^/ml in the same media. Adequate re-suspension at this step, by increasingly vigorous pipetting if necessary, was monitored by microscopy and was important for both dissociation of platelet aggregates and assay performance.

#### Platelet binding assay

Three days after injection of CFSE-labeled donor cells, lymph node cells were harvested and single cell suspensions prepared, depleted of sIg^+^ cells, split, one half held in I_10_ while the other half was pre-washed with EDTA as described above. Cells were re-suspended in I_10_ to 5x10^6^/ml, and placed on ice. Once CD41-APC-labeled and fixed platelets were prepared, lymph node cells were pelleted and re-suspended to 5x10^7^/ml in I_10_. Platelets (20μl at 5x10^8^/ml) and cells (20μl at 5x10^7^/ml) were combined in a 5 ml tube and incubated for 10 minutes at room temperature with gentle mixing every several minutes after which 380μl of a 1:1 mixture of I_10_ and H-B5 H10 Ca0.5 with 0.2 μg/ml PI was added. P-selectin dependent platelet binding to responding donor cells was assessed by comparing the extent of APC signal (anti-CD41-APC labeled platelets) acquired by donor activated T cells exposed to *P-selectin*^*+/+*^ vs *P-selectin*^*-/-*^ platelets. Data was acquired with gating for responding donor cells (CFSE-diluted) by CFSE signal level, and by excluding unbound platelets, large aggregates, and dead cells by gating against low forward-scatter events (platelets), high forward-scatter events (aggregates), and FL3^bright^ events (dead cells) respectively.

### Immobilized P-selectin-hIgG adherence assay

The cell adhesion assay applied was based on that previously described [12, 13] and modified as follows. Goat-anti-human IgG (Southern Biotech, 2040–01) was diluted to 5 μg/ml in pH 8.5 carbonate buffer (10 mM Na_2_CO_3_ and 35 mM NaHCO_3_), dispensed in 75μl volumes into V-bottom wells of 96-well polystyrene plates (Nunc 249662) and incubated for 2 hours at 37°C. Wells were washed 4x with blotto (5% skim milk powder in PBS with 0.01% sodium azide), incubated in blotto for 30 minutes at 37°C, and for 10 minutes at 37°C in BB (3% BSA in PBS). Wells were washed 2x with selectin binding buffer (SBB = 2 mg/ml BSA in H+), then incubated with 75μl per well of SBB ± 1 μg/ml P-selectin-hIgG chimera for 1 hour at room temperature and washed 4x with SBB prior to cell addition. Donor-derived activated T cells in adhesion assays were distinguished by CTV tracking dye labeling done prior to adoptive transfer. Lymph node cells containing donor derived activated T cells 3 days after adoptive transfer were prepared in I_10_, filtered through 70μm nylon mesh, and depleted of sIg^+^ cells with Dynabeads sheep anti-mouse IgG (Invitrogen 11031). Cells were re-suspended to 10^7^/ml and held on ice in I_10_ until used for adhesion assay. Just prior to assay, cells were diluted to 4x10^5^/ml cells in SBB + 15% FCS ± 10mM EDTA, aliquoted into prepared wells at 100μl/well and incubated for 15 minutes at 37°C. Plates were then spun for 10 minutes at 80g at room temperature with slow acceleration and minimal braking on deceleration. Nadirs from quadruplicate wells for each well group were individually harvested in 50μl drawn with a single channel P200 pipette and pooled into BSA-coated U-bottom wells. To remnants in each V-bottom well, 10μl of 50mM EDTA in H- B2 was added and mixed by light vortexing, incubated on ice for 4’, re-vortexed lightly for 5 seconds, re-incubated on ice for 4’, re-vortexed lightly for 5 seconds, and pelleted at 420g for 2 minutes. The supernatant was flicked out and the cell pellet re-suspended in 150μl H- B5 E2, transferred to BSA-coated U-bottom wells, transitioned to I_10_, stained with CD8-APC, and washed. Cell pellets were re-suspended with 120μl I_10_ containing PI and 5x10^3^ 10μm CML latex beads (Invitrogen C37259) used for counting. Cell counting by flow analysis was performed as described above using the LSRII and acquiring both beads and donor activated T cells based on light scatter and CTV florescence in triplicate counts of 10^3^ beads per sample.

## Results

### Discovery of PSL2

**Fig 1:**
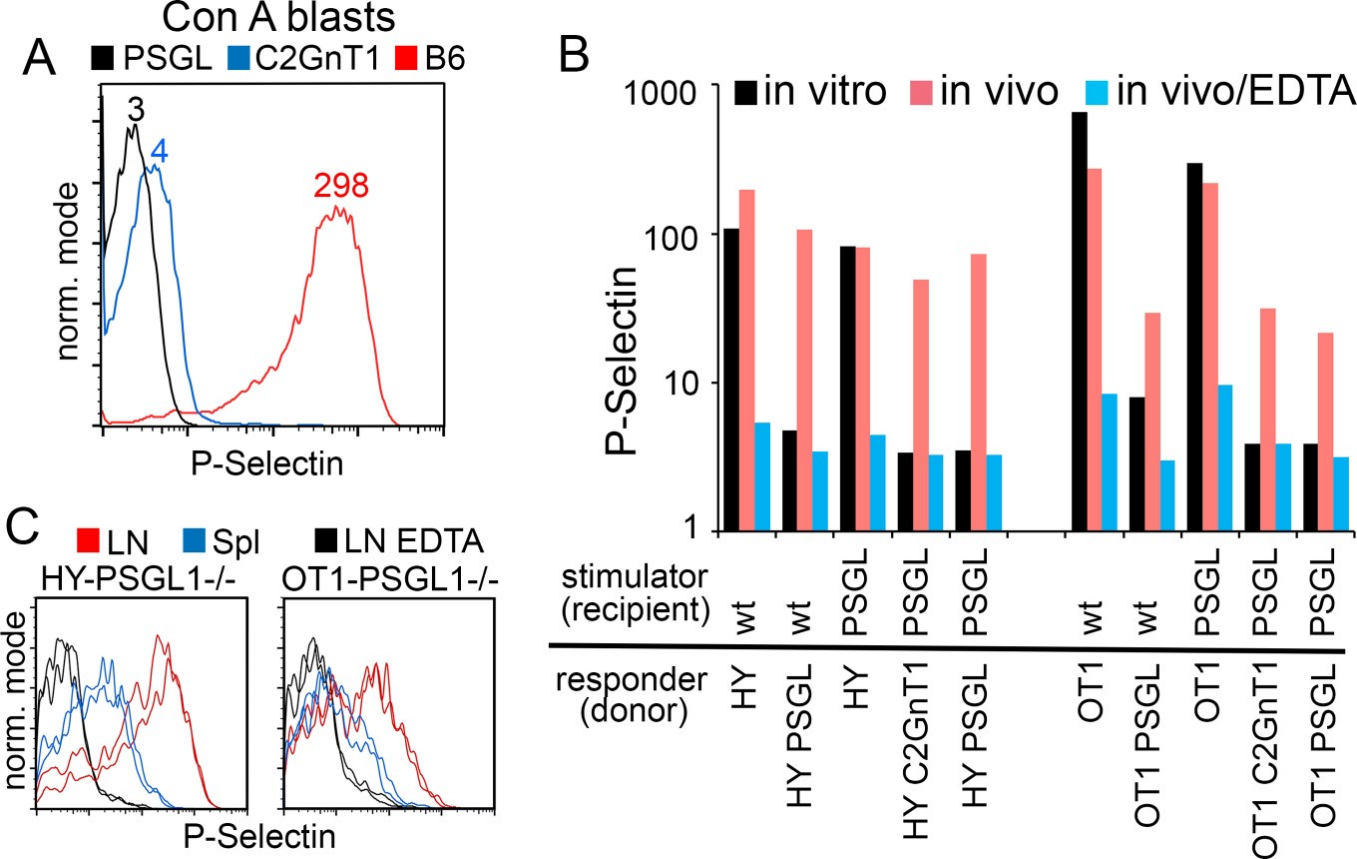
Detection of a PSGL1-independent P-selectin ligand on in-vivo activated T cells. (A) Formation of PSGL1-PSelL requires C2GnT1: Spleen cells from *B6*, *PSGL-1*^*null*^, or *C2GnT1*^*null*^ mice were activated in vitro with Concanavalin A for two days, sub-cultured with IL2 supplemented media for two additional days, and then stained with PSel-hIgG chimera. P-selectin geometric mean fluorescence values shown. Figure representative of more than nine independent analyses. (B) A PSGL1-independent P-selectin ligand is detected on CD8 T cells activated in vivo. Geometric mean P-selectin staining of day-3 activated HY male-antigen specific, or OT1 ovalbumin specific, T cell receptor transgenic CD8+ T cells responding to antigen in vitro or in vivo. Responding T cell receptor transgenic cells on wild type (*wt*), *PSGL-1*^*null*^, or *C2GnT1*^*null*^ genetic backgrounds were either stimulated in vitro with splenic dendritic cells (male or Ova-pulsed) from B6 (*wt*) or *PSGL-1*^*null*^ mice, or adoptively transferred into *wt* or *PSGL-1*^*null*^ male recipients. EDTA was included during P-selectin staining in parallel samples of in-vivo generated cells to confirm divalent cation (Ca^++^) dependent selectin binding. Flow analysis was gated for viable propidium iodide^negative^ (PI^neg^), CD8^+^, responding (CFSE-diluted) donor cells. The data shown are single sample staining values representing biological replicates for the two T cell receptor transgenic models examined. Fig 1B is representative of more than seven independent analyses. (C) PSL2 is preferentially displayed by CD8^+^ activated T cells responding in peripheral lymph node. P-selectin staining of CFSE-labeled *HY-PSGL*^*null*^ T cells responding in two *PSGL1*^*null*^ male recipients (left panel) or CFSE-labeled *OT1-PSGL*^*null*^ T cells responding to ovalbumin antigen in two *PSGL1*^*null*^ recipients (right panel). Donor cells were recovered from peripheral lymph nodes or spleen three days after adoptive transfer. Staining of duplicate recipient mice are shown for each donor. Control P-selectin staining in the presence of EDTA shown for respective donor/recipient combinations. Ex-vivo cells were subjected to gating for viable PI^neg^, CD8^+^, responding (CFSE-diluted) donor cells. Geometric mean P-selectin values for *HY-PSGL*^*null*^ (left panel) are LN EDTA 4/4, spleen 14/15, lymph node 82/111; for *OT1-PSGL*^*null*^ (right panel) LN EDTA 5/5, spleen 8/10, lymph node 24/27. Fig 1C is representative of three independent analyses.

The current paradigm for induction of P-selectin ligand on CD8^+^ T cells begins with their antigen-driven activation in secondary lymphoid organs and the subsequent induction of glycosyltransferases, including C2GnT1, that synthesize sLex-modified Core 2 branched O-glycans on PSGL1 [6, 14]. Data shown in Fig 1A illustrate P-selectin staining of CD8^+^ T cells after in vitro activation by mitogen and exemplify how P-selectin ligand expression is dependent on co-expression of PSGL1 and C2GnT1. As shown in Fig 1B, when antigen specific CD8^+^ T cells were activated by dendritic cells in vitro using two independent T cell receptor transgenic models, OT1 responses specific for ovalbumin and HY responses specific for male antigen, this pattern of PSGL1-PSelL formation is recapitulated precisely. Formation of P-selectin ligand on in-vitro activated CD8^+^ T cells was again dependent on T cells expressing both PSGL1 and C2GnT1. These observations were consistent with PSGL1 being the sole ligand for P-selectin on T cells activated in vitro. However, when these responses were generated in vivo after adoptive transfer of either OT1 or HY donor T cells in the presence of antigen, P-selectin staining of activated T cells was observed, even when responding T cells were deficient in PSGL1 or C2GnT1, Fig 1B. P-selectin staining was lost when EDTA was included in the staining procedure presumably reflecting involvement of Ca^2+^ in P-selectin’s carbohydrate recognition domain when binding ligand. These results suggested that a second P-selectin ligand (provisionally designated PSL2) was present on in-vivo activated CD8^+^ T cells.

To exclude PSGL1 and more clearly reveal PSL2 as P-selectin ligand in subsequent analysis, *HY* and *OT1* donor cells carrying either *PSGL*^*null*^ or *C2GnT1*^*null*^ alleles were used interchangeably in adoptive transfers. We previously noted that *C2GnT1*^*null*^ donors have superior lymph node access and gave superior activated T cells yields at day 3 compared to donor cells deficient in PSGL1 [15]. The use of *PSGL1*^*null*^ mice or *C2GnT1*^*null*^ mice as recipients in adoptive transfers also excluded the possibility of donor T cells acquiring decorated PSGL1 from stimulating dendritic cells through trogocytosis. Some trogocytosis of PSGL1 can occur in these systems but was a minor contribution to P-selectin ligand detected here (data not shown).

### PSL2 expression on activated T cells is prominent in lymph node but not spleen

PSL2 levels were compared on activated T cells harvested from lymph nodes and spleen in both OT1 and HY T cell receptor transgenic models as shown in Fig 1C. Surprisingly, the levels of PSL2 differed markedly on activated T cells from the two secondary lymphoid organs; donor cells responding in lymph node consistently exhibited higher levels of PSL2 than those harvested from spleen. PSL2 levels on activated T cells responding in mesenteric lymph node were intermediate between that seen on responding donor cells from other lymph nodes and spleen (not shown). We therefore focused analysis on T cells activated in peripheral lymph nodes.

### PSL2 expression is dependent on the presence of divalent cation

**Fig 2:**
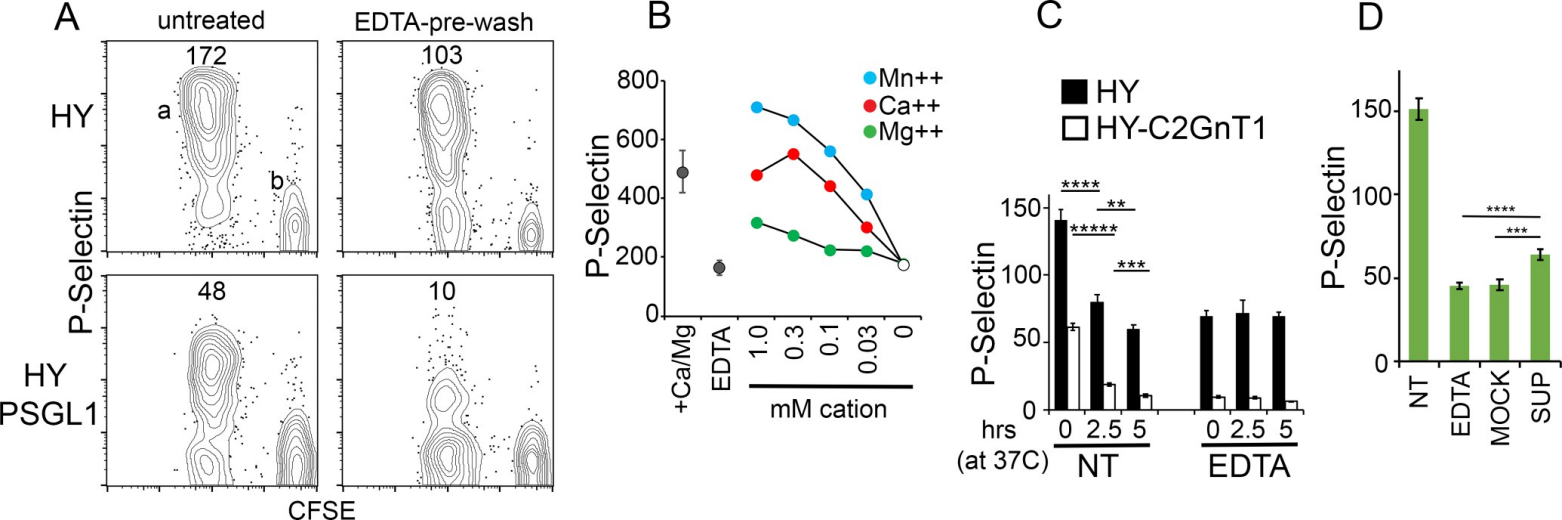
PSL2 association with activated T cells requires Ca^++^. (A) Cation removal eliminates expression of PSL2. P-selectin staining of ex-vivo CD8^+^ CFSE-labeled *HY* or *HY-PSGL1*^*null*^ donor T cells responding (CFSE^low^ cloud ‘a’) and non-responding (CFSE^high^ cloud ‘b’) at day 3 in *PSGL1*^*null*^ recipients (left panels). In right panels, aliquots of the same cells were washed twice in EDTA-containing media, returned to Ca^++^ replete media, and then stained with P-selectin. Geometric mean values for P-selectin staining of responding (CFSE diluted) donor cells are shown. Fig 2A is representative of five independent analyses. (B) Cation specificity to support PSL2 expression. *OT1-C2GnT1*^*null*^ cells responding to Ovalbumin in *PSGL1*^*null*^*Thy1*.*1* recipients were recovered at day 3. The cation-specific dependence of PSL2 expression on these cells was assessed after 5x serial washing in media containing the indicated concentrations of Mn^++^, Ca^++^, or Mg^++^ divalent cations, and returned to Ca^++^/Mg^++^ replete media, prior to P-selectin staining. Positive control, (+Ca/Mg), P-selectin staining of responding cells maintained in Ca^++^/Mg^++^ replete media. Negative control (EDTA) P-selectin staining of responding cells pre-washed with EDTA-media. Standard deviation of triplicate control stains shown. Fig 2B is representative of four independent analyses. (C) Persistence of PSGL1 P-selectin ligand and loss of PSL2 P-selectin ligand with in vitro culture. *HY* and *HY-C2GnT1*^*null*^ donor cells responding in independent male *PSGL1*^*null*^ recipients were harvested on day 3 and either untreated (*NT*), or pre-washed with EDTA-containing media (PSL2 stripped) and then transferred into Ca^++^/Mg^++^ replete media (EDTA). Cells in NT or EDTA groups were then held on ice (0), or cultured at 37°C for 2.5 or 5 hours prior to P-selectin staining. Mean and standard deviation of three P-selectin staining replicates for each condition is shown. P-values determined by two-tailed Student’s T-tests with equal variance; **** P value = 0.0005, ***** P value = 0.00001, *** P value = 0.001, ** P value = 0.008. Fig 2C is representative of three independent analyses. (D) PSL2 rebinding. CD8^+^ *HY-C2GnT1*^*null*^ donor T cells were recovered from peripheral lymph nodes of male *PSGL1*^*null*^ *Thy1*.*1* recipients on day 3 and semi-purified (to >50% purity). Responding donor derived cells were either stained for P-selectin *(*NT) or treated with EDTA-pre-wash to generate donor cells lacking PSL2 (EDTA) and a supernate extract. The supernate extract was made Ca^++^ replete and compared with a mock supernate, prepared identically except never exposed to cells, for their respective capacity to restore P-selectin ligand expression (SUP vs MOCK) on responding, EDTA pre-washed, donor-derived T cells. Mean and standard deviation of three P-selectin staining replicates for each condition is shown along with P-values determined by two-tailed Student’s T-test. **** P value = 0.00073, *** P value = 0.0018. Fig 2D is representative of eight independent analyses.

CD8^+^ T cells from *HY* mice (able to express both PSGL1 and PSL2 P-selectin ligands) or from *HY-PSGL1*^*null*^ mice (able to express only PSL2) responded in male recipients by proliferation and expression of P-selectin ligand as shown in Fig 2A. CFSE diluted populations were present in all four panels and exemplified for HY responders by population ‘a’. A subset of CD8^+^ cells in the HY model, population ‘b’, express endogenous (non-transgenic) T cell receptor alpha chains, do not respond to male antigen, remain CFSE^high^, and do not display P-selectin ligand; these non-responding donor cells serve as a reference point for P-selectin staining and proliferation (CFSE dilution). Notably, expression of both PSGL1 and PSL2 P-selectin ligand required prior T cell activation/proliferation. Surprisingly, brief exposure to EDTA-containing media at 4°C prior to staining with P-selectin rendered activated T cells devoid of PSL2 expression, even if returned to Ca^++^ replete conditions (EDTA-pre-wash). Pre-washing with calcium chelator EGTA also resulted in loss of PSL2 detection with P-selectin (data not shown). The EDTA pre-wash treatment was distinguished from controls (eg. Fig 1B) where EDTA was included during P-selectin staining to demonstrate calcium-dependent binding by selectin. In the latter case, EDTA prevents selectin binding to ligand by stripping Ca^++^ from the ligand-binding face of selectin, thereby disabling Ca^++^ ion mediated coordination between selectin and glycan atoms presented by ligand [16]. EDTA pre-wash will not remove PSGL-1 from cells as it is anchored via a transmembrane domain whereas PSL2 displayed on activated T cells was washed off. Thus the P-selectin signal remaining on responding *HY* T cells after EDTA pre-wash (Fig 2A upper right panel) could be ascribed to PSGL1, a signal absent on *HY-PSGL1*^*-/-*^ T cells (lower right panel). As shown in Fig 2B PSL2 expression was maintained relatively well when activated T cells were serially washed in minimal media with diminishing concentrations of Ca^++^ or Mn^++^ but was lost in media supplemented only with Mg^++^ divalent cation.

The Ca^++^ dependence of PSL2 detection might reflect either (i) a cation requirement for a conformational domain on PSL2 used in P-selectin binding, or (ii) a cation requirement for PSL2 association with the cell surface. Ca^++^ could mediate PSL2 binding directly to cell surface lipids, as for Gal-domain containing proteins, or through an alternate docking molecule on the T cell surface. When donor-derived activated T cells were harvested from lymph node and cultured briefly at 37°C, expression of PSL2 was lost while PSGL1 P-selectin ligand was maintained, Fig 2C. When activated T cells were pre-washed with EDTA-media and then cultured in Ca^++^ replete media, PSGL1 P-selectin ligand was maintained whereas PSL2 was lost in the initial wash and no recovery of PSL2 was observed. If PSL2 consisted of a conformational P-selectin ligand dependent on Ca^++^, recovery of PSL2 expression during culture might have been expected but did not occur.

If treatment with EDTA eluted PSL2 from the cell surface into media, the eluted supernatant might restore PSL2 expression if ‘stripped’ activated T cells were subsequently re-exposed to the supernatant in the presence of Ca^++^. As shown in Fig 2D this appeared to be the case as partial restoration of PSL2 expression relied on provision of both Ca^++^ and supernate. The low, but significant PSL2 re-binding signal exemplified by ‘SUP’ when compared to MOCK and the positive control NT, likely reflected a low concentration of PSL2 available for re-binding in EDTA-eluted supernatants re-supplemented with calcium. Based on these data we conclude that PSL2 is a P-selectin ligand bound through Ca^++^ to the surface of activated T cells. EDTA pre-washing strips PSL2 from activated T cells while leaving PSGL1 expression intact.

### PSL2 on activated T cells is cell-extrinsic and dependent on recipient C2GnT1 expression

**Fig 3:**
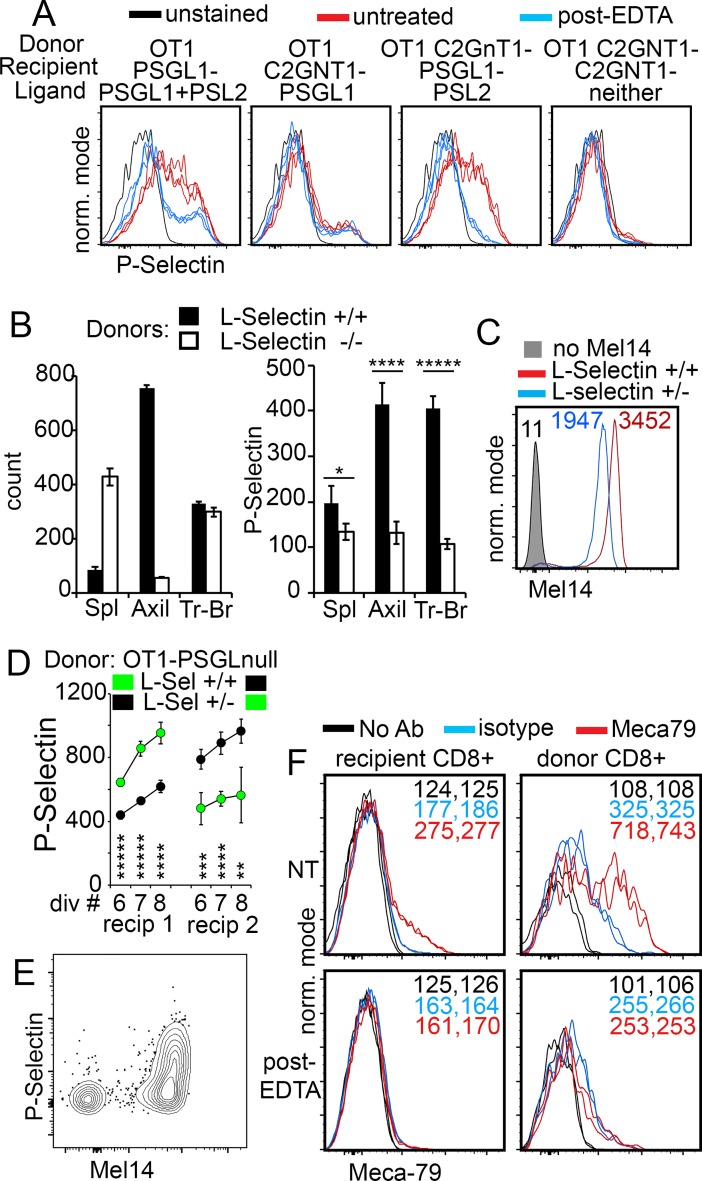
PSL2 is T cell-extrinsic, dependent on recipient C2GnT1 expression, and docks on L-selectin. (A) PSL2 display requires C2GnT1 expression in recipient and, together with PSGL1, constitute the total P-selectin ligand on responding donor-derived CD8^+^ T cells. *OT1* or *OT1C2GnT1*^*null*^ donor cells were transferred with ovalbumin into *PSGL1*^*null*^ or *C2GnT1*^*null*^ recipients and analyzed on day 3. P-selectin staining was compared on these (untreated) cells and on corresponding EDTA-pre-washed samples (*post-EDTA*). The anticipated ligands ‘PSelL’ contributing to P-selectin binding based on data shown above is indicated. Triplicate P-selectin stains for each sample are plotted. Fig 3A shown is representative five independent analyses. (B) L-selectin-independent entry of CD8 T cells into tracheo-bronchial LN. *OT1-PSGL*^*null*^*-LSel*^*+/+*^ and *OT1-PSGL*^*null*^*-LSel*^*-/-*^ donor cells labeled with CTV or CFSE tracking dyes were co-injected iv into *PSGL-Thy1*.*1* recipients together with ovalbumin. On day 3, spleen, axillary lymph nodes, and tracheo-bronchial lymph nodes were separately harvested and assessed for relative numbers of donor-derived responding T cells (left column graph) and their expression of P-selectin ligand (right column graph). Mean and standard deviation of triplicate samples used for counting and P-selectin staining for each condition is shown. P-values for comparing levels of P-selectin staining of L-Sel^+/+^ vs LSel^-/-^ donor cells were determined by two-tailed Student’s T-tests with equal variance; * P value = 0.07, **** P value = 0.0007, ***** P value = 0.00006. Fig 3B shown is representative of three independent analyses. (C) L-selectin expression is gene dose dependent. Mel 14 (anti-L-selectin) staining of naïve CD8^+^ T cells from peripheral lymph node of *OT1-PSGL1*^*-/-*^*LSel*^*+/-*^ versus *OT1-PSGL1*^*-/-*^*LSel*^*+/+*^ mice. Geometric mean P-selectin values are indicated. (D) PSL2 expression on activated CD8^+^ T cells responding in PerLN correlates with L-selectin gene dose. Naïve *OT1-PSGL1*^*-/-*^*LSel*^*+/+*^ and *OT1-PSGL1*^*-/-*^*LSel*^*+/-*^ (L-selectin heterozygous) donor cells were labeled with CFSE or CTV tracking dyes, combined, and co-injected with ovalbumin into *PSGL1*^*-/-*^ *Thy1*.*1* recipients. P-selectin staining is shown at comparable cell division numbers for donor cells obtained from the same recipient at day 3. Recipient #1, *OT1-PSGL LSel*^*+/+*^ (CFSE, green) and *OT1-PSGL1 LSel*^*+/-*^ (CTV, black). Recipient #2, same donor cells but dyes reversed. Mean and standard deviation of geometric mean P-selectin staining at comparable division number is shown. P-values for comparing levels of P-selectin staining at each division number were determined by two-tailed Student’s T-tests with equal variance; recipient #1: division #6 P value = 0.00003 *****, division #7 P value = 0.00001*****, division #8 P value = 0.0001****; recipient #2: division #6 P value = 0.002 ***, division #7 P value = 0.0002****, division #8 P value = 0.05** Fig 3D shown is representative of three independent analyses. (E) Mel14 and P-selectin co-staining of activated CD8^+^ T cells. CFSE-labeled donor *OT1C2GnT1*^*-/-*^ cells were transferred with ovalbumin into *PSGL1*^*-/-*^ recipients. Responding donor-derived cells in peripheral lymph node were assessed at day 3 for P-selectin binding and L-selectin expression; analysis gated on responding (CFSE-diluted), viable, CD8^+^ donor cells. Fig 1E is representative of five independent analyses. (F) Meca 79 staining of CD8^+^ recipient cells versus donor-derived activated T cells. CTV-labeled *OT1C2GnT1*^*-/-*^ donor cells were transferred into *PSGL1*^*-/-*^ recipients with ovalbumin. On day 3 both recipient-derived CD8^+^ T cells and responding (CTV diluted), donor-derived, CD8^+^ cells were stained for Meca79 expression either before (top panels) or after (bottom panels) applying EDTA-wash conditions used to strip PSL2. Geometric mean P-selectin staining values for technical replicates are shown. Fig 3F is representative of three independent analyses.

Data in Fig 1 indicated that PSL2 expression on in vivo generated activated T cells was independent of cell-intrinsic C2GnT1—in contrast to P-selectin recognition of PSGL1. Our initial interpretation of this difference was that the structure of the ligand on PSL2 must differ from the glycan array presented on PSGL1 for P-selectin recognition. However, when activated T cells were generated in recipients lacking *C2GnT1*, P-selectin detection of PSL2 was lost as shown in Fig 3A. This finding suggested that the requirement for C2GnT1 in P-selectin recognition of PSL2 was indeed preserved and that PSL2 displayed by donor activated T cells was sourced from the recipient and not a cell-intrinsic product of responding donor T cells. Sourcing of PSL2 from recipient tissue was also consistent with the observations that (i) T cells activated in vitro lack PSL2 (Fig 1A and 1B), (ii) the association of PSL2 with activated T cells was Ca^++^ dependent and reversible, (Fig 2A, 2B and 2D), and (iii) PSL2 expression was rapidly and irreversibly lost during culture of ex-vivo activated T cells, a loss accelerated by brief washing in EDTA-media (Fig 2C). Collectively these data demonstrated that PSL2 on activated T cells was cell-extrinsic and that the total P-selectin ligand generated on activated T cells during the primary response of OT1 CD8^+^ T cells in lymph node was the sum of the cell-intrinsic PSGL1 and cell-extrinsic PSL2; no additional ligand of P-selectin was apparent in this system (Fig 3A).

### PSL2 on activated T cells is docked on L-selectin

Since there is some overlap in P-selectin and L-selectin ligand recognition, the cell-extrinsic source of PSL2 and preferential detection of PSL2 on activated T cells developing in lymph node vs spleen suggested that PSL2 might be a soluble ligand of L-selectin produced in lymph nodes. With this background, a primary candidate for PSL2 became Glycam-1, a secreted L-selectin ligand produced by HEV [17, 18] that can also bind immobilized P-selectin [19]. The *Glycam1*^*null*^ mouse was previously generated but abandoned when its physiological role as an L-selectin ligand could not be confirmed. We therefore re-derived and tested *Glycam1*^*null*^ mice for deficiency in loading PSL2 on activated T cells but observed no defect thus excluding Glycam1 as a candidate for PSL2 (S1 Fig). Using locally available knockout mice, we formally eliminated other L-selectin ligands beyond PSGL1 and Glycam-1 as PSL2 candidates; these include CD34, CD44, endogycan, podocalyxin (data not shown).

However, several observations indicated that L-selectin on activated T cells was indeed the dock for PSL2. First, *L-selectin* ^*-/-*^ T cells access most lymph nodes poorly, preventing intra-lymph node comparisons with *L-selectin*^*+/+*^ cells, but it has been reported that they can access mediastinal lymph nodes more efficiently [20]. We found that entry of *L-selectin*^*-/-*^ donor T cells into the tracheobronchial lymph node [21] did occur and that these nodes were a relatively good source of donor-derived activated T cells. Tracking dyes were used to distinguish donor *OT1*-*PSGL1*^*-/-*^ *L-selectin* ^*+/+*^ and *OT1*-*PSGL1*^*-/-*^ *L-selectin* ^*-/-*^ activated T cells responding in the same tracheobronchial lymph nodes of *PSGL1*^*null*^ recipients and to assess the effect of L-selectin deficiency on PSL2 loading. As shown in Fig 3B, both *L-selectin* ^*+/+*^ and *L-selectin* ^*-/-*^ donor cells could access the tracheobronchial nodes in equal numbers but PSL2 was not loaded significantly on *L-selectin* ^*-/-*^ activated T cells whereas *L-selectin* ^*+/+*^ activated T cells responding in the same nodes loaded PSL2 as well as that seen in axillary lymph nodes.

Second, *L-selectin* ^*+/-*^ T cells express approximately half the levels of cell surface L-selectin relative to their *L-selectin* ^*+/+*^ counterparts (Fig 3C) and access lymph nodes less efficiently [22, 23]. Whether such a two-fold difference in L-selectin expression would influence PSL2 loading on activated T cells was investigated. PSL2 loading onto responding *L-selectin* ^*+/-*^ T cells and co-injected *L-selectin* ^*+/+*^ T cells was compared after harvest from the same peripheral lymph nodes. The results exemplified in Fig 3D demonstrated that, at comparable division numbers, reduced expression of L-selectin was indeed paralleled by reduced PSL2 loading.

Third, L-selectin can be cleaved from the cell surface after T cell activation and, if required for PSL2 docking, such loss of L-selectin would be incompatible with PSL2 detection. The status of L-selectin expression and PSL2 display on donor-derived activated T cells was therefore assessed to further document the relationship between L-selectin and PSL2. As shown in Fig 3E, a fraction of donor-derived activated T cells were L-selectin^low/negative^, presumably due to L-selectin shedding, while a second subset remained L-selectin ^high^; PSL2 was only detected on a portion of these L-selectin^high^ cells. Given MEL-14 specificity for the ligand-binding region on L-selectin, the same region that presumably binds PSL2, one might ask how MEL-14 could detect L-selectin if PSL2 were present? We reconciled our capacity to detect both PSL2 (with P-selectin-hIgG) and L-selectin (with MEL-14) as shown in Fig 3E with a reasoned speculation that after T cell activation either a fraction of L-selectin remains unable to bind PSL2, as on naïve cells, and/or that PSL2 availability is limiting for loading onto L-selectin. In summary, this data demonstrated that activated T cells lacking L-selectin expression also lack PSL2 display while PSL2 is displayed on a subset of L-selectin^high^ activated CD8^+^ T cells.

Fourth, if PSL2 were a de facto ligand of L-selectin, reagents that detect L-selectin ligand might reveal PSL2 displayed by activated T cells. One such L-selectin ligand-specific reagent is the IgM monoclonal antibody Meca-79[24]. Meca-79 identifies most L-selectin ligands as it binds 6-sulfo sLex on extended core 1 O-glycans[25] but not 6-sulfo-sLex L-selectin ligand on core 2 (C2GnT1) branched O-glycans or N-glycans [26]. We therefore examined if Meca-79 would bind activated T cells. Results shown in Fig 3F demonstrated that such specific binding was indeed detected. Moreover, the Meca79 signal was lost if cells were pre-treated with the same EDTA washing procedure used to effectively remove PSL2.

Fifth, PSL2 association with activated T cells requires Ca^++^, consistent with the requirement for this cation in L-selectin binding its ligands via the lectin domain.

On the basis of the above observations we conclude that L-selectin is the dock for PSL2 on activated T cells and that PSL2 is a ligand for both L-selectin and P-selectin that can be simultaneously engaged by both selectins.

### Selectin recognition of PSL2

Whether PSL2 could serve as a ligand for E-selectin or L-selectin was also explored. As shown in S2 Fig, PSL2 was bound to some degree by all selectins and binding was eliminated by EDTA pre-washing. Selectin recognition requires involvement of sialic acid in sLex presented on their respective ligands. As shown in S3 Fig, either P-selectin recognition of PSL2, and/or PSL2 docking onto L-selectin, were vulnerable to neuraminidase indicating that sialic acid was indeed required for one, or both, of these interactions.

### PSL2 and PSGL1 co-operate in activated T cell adhesion to activated platelets and to immobilized P-selectin

Data presented above show that PSL2 is bound to L-selectin on activated T cells and that this complex could be recognized by P-selectin in solution. Physiological P-selectin is expressed on the luminal surface of activated endothelium and on activated platelets to initiate formation of adhesive tethers with circulating leukocytes. We therefore assessed if the PSL2/L-selectin complex would support activated T cell adhesion to activated platelets and immobilized P-selectin. We also sought to determine how PSL2 might cooperate with PSGL1 in these physical-adhesive interactions. The donor-derived activated T cells of interest that expressed PSL2 comprised approximately 1% of total recipient lymph node cells. To assess adhesion of these ‘rare’ cells we used two flow cytometer-based selectin adhesion assays, both of which could be applied with minimal specialized infrastructure. Previously published procedures to analyze cell adhesion to platelets [27, 28] or immobilized P-selectin [12, 13] were adapted to assess adhesion of murine activated T cells to these substrates.

**Fig 4:**
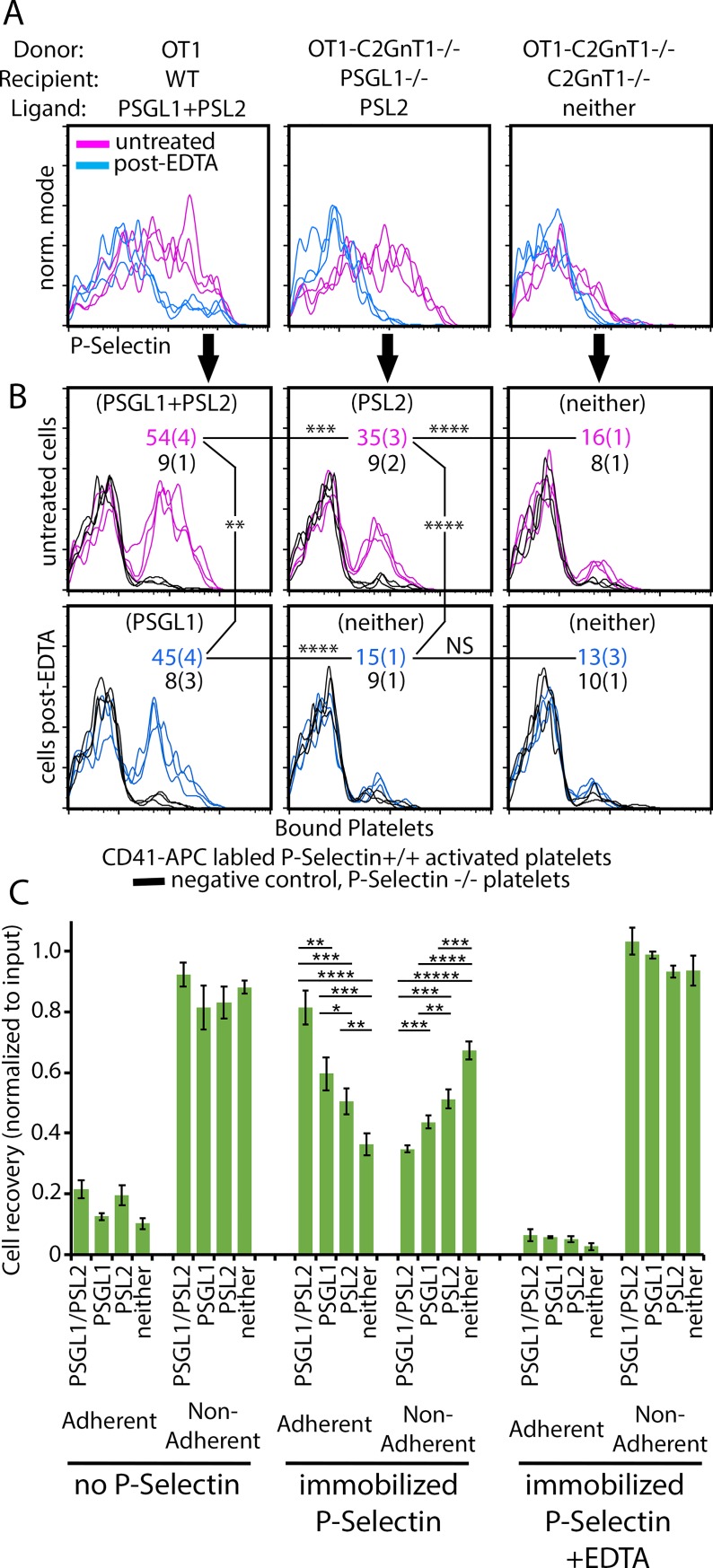
PSL2 is an adhesion receptor. (A) Activated T cells expressing PSGL1 and/or PSL2. P-selectin binding profiles of activated T cells generated using donor/recipient combinations specified expressing PSGL1 and PSL2, PSL2 alone, or neither. Staining with P-selectin was performed on untreated cells or on cells after EDTA pre-washing revealing the independent contributions of PSGL1 (EDTA-resistant) and PSL2 (EDTA-strippable). Independent triplicate staining profiles are shown. Fig 4A is representative of five independent analyses. (B) Activated platelets expressing P-selectin can bind activated T cells via PSGL1 and/or PSL2. Platelets from *P-selectin*^*+/+*^ and *P-selectin*^*-/-*^ mice were isolated, labeled with anti-CD41-APC, activated with thrombin, paraformaldehyde fixed, and washed. Cells shown in (A) were then assessed for capacity to bind CD41-APC labeled platelets by flow cytometry. Free platelets were excluded from analysis by forward/side light scatter characteristics. Platelets from *P-selectin*^*-/-*^ mice were assayed in parallel to resolve P-selectin dependent platelet adhesion to responding T cells. Expected P-selectin ligand displayed in parentheses. Mean and standard deviation of percentage CD41-APC+ (platelet bound) cells indicated for *P-selectin*^*+/+*^ platelets and *P-selectin*^*-/-*^ platelets. P-values for differences in *P-selectin*^*+/+*^ platelet binding determined by Student’s T test, with confidence levels reaching 2, 3 or 4 SD as indicated by **,***,**** respectively. Fig 4B is representative five independent analyses. (C) PSL2 supports adhesive interaction with immobilized P-selectin. Tracking dye-labeled donor T cells responding in *OT1→ PSGL*^*null*^, *OT1→ C2GnT1*^*null*^, *OT1 C2GnT1*^*null*^*→ PSGL*^*null*^, and *OT1 C2GnT1*^*null*^ *→ C2GnT1*^*null*^ adoptive transfers yielded activated cells expressing PSGL1 and PSL2, PSGL1, PSL2, or neither ligand respectively. These cells were compared for adhesion to P-selectin immobilized on V-bottom wells under shear generated by centrifugation. Wells were coated with blocking agent alone (no P-selectin) or with PSel-hIgG chimera (immobilized P-selectin). Cells were also centrifuged in wells pre-coated with P-selectin but in the presence of EDTA to prevent selectin mediated binding (immobilized P-selectin + EDTA*)*. Non-adherent cells pelleting to the nadir were harvested directly and cells adhering to the tapered well sides were subsequently harvested using EDTA elution (Adherent). Donor derived activated T cells in non-adherent vs adherent fractions were quantified by flow cytometry-based counting and each normalized to input cell numbers. Triplicate counts of pooled quadruplicate samples were used to generate mean and standard deviation values of cell yields as shown. The significance of differences in cell yields were determined by applying two-tailed Student’s T-tests with equal variance. P-values reaching 1–5 sigma levels of significance were indicated by *, **, ***, ****, ***** respectively. The results shown are representative of four independent analyses.

In the first assay, thrombin-activated murine platelets were assessed for P-selectin dependent binding to activated T cells that expressed PSGL1 and PSL2, PSL2 alone, or neither ligand. An aliquot of each cell preparation was subjected to EDTA pre-washing to resolve the contribution of PSL2 to platelet binding. P-selectin staining controls shown in Fig 4A illustrate how PSGL1 (EDTA-pre-wash resistant) and PSL2 (EDTA-pre-wash vulnerable) constituted the total P-selectin ligand expressed by the activated T cells. By comparing adhesion of wild type (*wt*, *P-selectin*^*+/+*^) platelets versus platelets obtained from *P-selectin*^*-/-*^ mice, the involvement of P-selectin in platelet adhesion to activated T cells was resolved. Data shown in Fig 4B indicated that PSL2 was able to support P-selectin-dependent adhesion between activated T cells and activated platelets, and that PSL2 co-operated with PSLG1 in additive manner. EDTA pre-washing of activated T cells expressing only PSL2 eliminated platelet binding—there was very little background platelet binding when both PSGL1 and PSL2 were absent or when *P-selectin*^*-/-*^ platelets were used. We therefore conclude that PSL2 can provide adhesive support with PSGL1 for physical interaction between activated T cells and P-selectin on activated platelets.

In the second adhesion assay, the capacity of the PSL2/L-selectin complex to support activated T cell adhesion was also assessed by its ability to retard activated T cell rolling on a P-selectin coated surface. The method used to assess adherence entailed centrifuging activated T cells in V-bottom wells coated with P-selectin, centrifugal force providing shear against selectin engagement. Non-adherent cells pelleted to the nadir were harvested and then adherent cells retained on the tapered well walls were harvested separately by elution with EDTA. Donor-derived T cells recovered in both fractions were quantified by flow cytometry. Results using this assay shown in Fig 4C demonstrated that both PSGL1 and PSL2 contributed to activated T cells adherence in additive manner; there was no indication of adherence synergy between PSGL1 and PSL2 or a dominant contribution by either ligand. Activated T cell adhesion required immobilized P-selectin and divalent cation insofar as adhesion was prevented when EDTA was included. Results also suggested that a minor component of adherence to immobilized P-selectin detected in this assay was independent of both PSGL1 and PSL2 insofar as activated T cells lacking both PSL2 and PSGL1 adhered above background defined in wells lacking P-selectin. We therefore conclude that PSL2 can provide adhesive support with PSGL1 for physical interaction between activated T cells and either activated platelets or immobilized P-selectin.

### PSL2 transfer from recipient lymph node cells to activated CD8^+^ T cells

**Fig 5:**
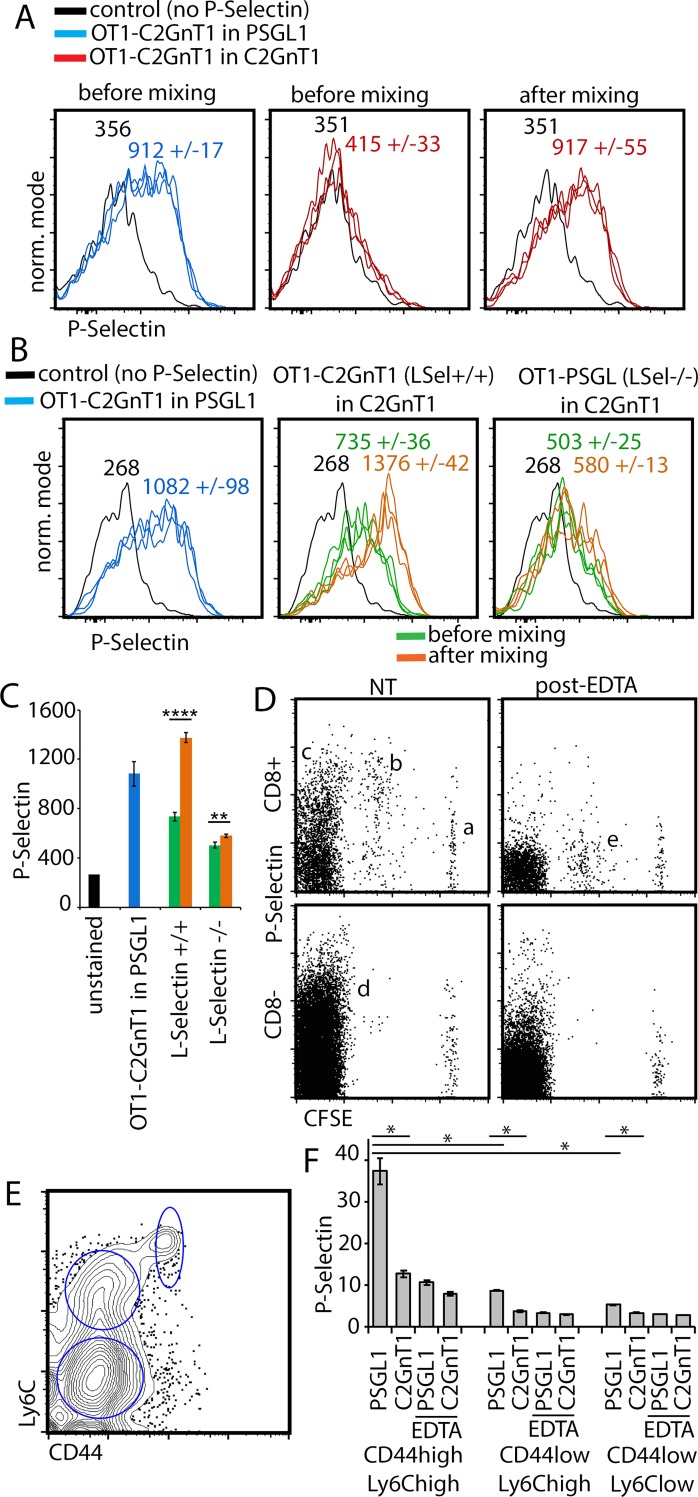
PSL2 transfer from resident lymph node cells to activated T cells. (A) CD8^+^ T cells responding in C2GnT1^null^ recipients lack PSL2 but can be stained with P-selectin after in vitro mixing with C2GnT1^+^ lymph node cells. *OT1C2GnT1*^*null*^ donor cells responding in peripheral lymph node of *PSGL1*^*null*^ recipients at day 3 were stained with P-selectin to detect PSL2 (left panel). The same donor cells responding in *C2GnT1*^*null*^ recipients fail to stain with P-selectin (center panel, ie. lack PSL2) but can be stained with P-selectin (right panel) after mixing with whole peripheral lymph node cells from the *OT1C2GnT1*^*null*^ *→ PSGL1*^*null*^ adoptive transfer (re: Methods). The contribution of recipient:donor, or donor:donor, doublets was excluded during flow cytometry by use of doublet discrimination and distinct fluorescent tracking dyes for each responding donor cell preparation. Triplicate P-selectin staining is shown along with the average geometric mean fluorescence and standard deviation. Fig 5A is representative of five independent analyses. (B) L-selectin expression on activated CD8^+^ T cells is required for acquisition of P-selectin ligand. P-selectin staining of *OT1-C2GnT*^*-/-*^ *(L-selectin*^*+/+*^*)* CD8^+^ T cells responding in peripheral lymph node of *PSGL1*^*null*^ recipients (*OT1C2GnT1*^*null*^ *L-selectin*^*+/+*^ *→ PSGL1*^*null*^) detects PSL2 (left panel). Center panel: P-selectin staining of donor T cells responding in spleen after adoptive transfer of *OT1-C2GnT1*^*null*^ *L-selectin*^*+/+*^ *→ C2GnT1*^*null*^ (‘PSL2-deficient’), before and after mixing/pelleting with whole peripheral lymph node cells from the ‘PSL2-competant’ *OT1C2GnT1*^*null*^ *L-selectin*^*+/+*^ *→ PSGL1*^*null*^ adoptive transfer. P-selectin staining is enhanced after mixing when responding CD8^+^ donor T cells are *L-selectin*^*+/+*^ (center panel) but not when they are *L-selectin*^*-/-*^ (right panel). As in Fig 5A, the potentially confounding possibility of recipient:donor, or donor:donor, doublet formation in the analysis was excluded during flow cytometry using doublet discrimination and since each responding donor cell preparation had been labeled with distinct fluorescent tracking dyes. Triplicate P-selectin staining is shown along with the average geometric mean fluorescence and standard deviation. Fig 5B is representative of four independent analyses. (C) Graphical summary of geometric mean P-selectin staining from flow cytometry data shown in Fig 5B. with corresponding color code. Standard deviation of triplicate stains shown by error bars. The significance of differences in P-selectin staining of responding donor-derived *LSel*^*+/+*^ and *LSel*^*-/-*^ CD8+ T cells from spleen were determined before and after mixing with ‘PSL2-competant’ cells by applying two-tailed Student’s T-tests. P-values reaching 4 (****, P value .00004) and 2 (**, P value .01) sigma levels of significance are indicated. (D) P-selectin ligand detected on *resident* lymph node cells is removed with EDTA. P-selectin staining of whole lymph node cells 3 days after adoptive transfer of CFSE-labeled donor *HY-C2GnT1*^*null*^ into a male *PSGL*^*null*^ recipient. CD8^+^ and CD8^-^ cells of recipient and donor origin were analyzed either directly (NT) or after applying the same EDTA washing conditions used to strip PSL2 (post-EDTA). As also shown in Fig 2A, responding CFSE^low^ donor cells Fig 5D (b) express PSL2 while non-responding CFSE^high^ donor cells Fig 5D (*a*) lack it. A fraction of recipient CFSE-negative CD8^+^ and CD8^-^ lymph node cells (c) and (d) respectively, were stained with P-selectin (left panels). P-selectin staining of these recipient cells was lost if pre-washed with EDTA (post EDTA, right panels), as observed for donor-derived activated T cells (subset ‘b’ versus ‘e’. Fig 5D is representative of more than three independent analyses. (E) Memory phenotype CD8^+^ cells in ‘naïve’ peripheral lymph node cells. Gated analysis of the CD8^+^ peripheral lymph node cells from untreated *PSGL1*^*null*^ mice for expression of CD44 and Ly6c. Fig 5E is representative of three independent analyses. (F) Within the CD8^+^ subset of untreated lymph node cells, only memory phenotype cells express PSL2. P-selectin staining of CD8^+^ lymph node cells with gating as shown in Fig 5E for CD44^high^Ly6c^high^, CD44^low^Ly6c^high^, and CD44^low^Ly6c^low^ subsets from naïve *PSGL1*^*null*^ or *C2GnT1*^*null*^ mice, either before or after EDTA pre-washing (EDTA). Standard deviation of triplicate stains shown by error bars. The significance of differences in P-selectin staining of the indicated CD8^+^ T cell subpopulations were determined by applying two-tailed Student’s T-tests with equal variance. P-values for specific comparisons were marked (* indicating P values ≤ 0.0001). Fig 5F is representative of three independent analyses.

The cellular source of PSL2 is unknown but likely to be high endothelial cells (HEC) inasmuch as these cells synthesize L-selectin ligands and are present in lymph nodes but absent in spleen. Understanding the mechanics of how PSL2 is acquired by activated T cells would contribute to the physiological framework of this selectin ligand. Despite the limited tools currently available, some unexpected insights into PSL2 acquisition were revealed in the course of our studies. In experiments with activated T cells expressing different combinations of PSGL1, PSL2, or neither, we observed that when ex-vivo cell suspensions of activated T cells lacking PSL2 were mixed with PSL2-expressing cells in vitro, the former rapidly acquired PSL2. As shown in Fig 5A, activated T cells from *OT1-C2GnT1*^*null*^ donors responding in *PSGL1*^*null*^ recipients expressed PSL2 whereas the same donor cells responding in *C2GnT1*^*null*^ recipients did not. However, when lymph node cell suspensions from these two recipients were mixed, activated T cells from *C2GnT1*^*null*^ recipients could then be stained with P-selectin. If the acquired P-selectin ligand was indeed PSL2, this observation suggested that once activated, T cells enter a ‘PSL2-receptive’ state where PSL2 can be supplied by resident lymph node cells and that L-selectin would likely be required for its docking on receptive T cells. To evaluate the role of L-selectin in this transfer, *L-selectin*^*-/-*^ and *L-selectin* ^*+/+*^ T cells were activated in *C2GnT1*^*null*^ recipients (‘PSL2-deficient’ environments) and isolated from spleen thereby yielding ‘PSL2-receptive’ T cells that differentially expressed L-selectin. These cells were then compared for their ability to acquire P-selectin staining after mixing with ‘PSL2-competant’ lymph node cells. The results shown in Fig 5B and 5C demonstrated that L-selectin was required for acquisition of the P-selectin ligand. These results reinforced the view that the transferred P-selectin ligand was indeed PSL2 and that PSL2 can be acquired by L-selectin on activated T cells through cell:cell contact. Since lymph node cell suspensions collected by our methods would not likely contain HEC, the source of in-vitro transferred PSL2 was most likely lymph node-resident lymphocytes themselves, leading directly to the question of what resident cells in lymph node cell suspensions could supply PSL2 to activated T cells.

P-selectin staining of lymph node cells three days after adoptive transfer of CFSE-labeled *HY-C2GnT1*^*null*^ donor cells into male *PSGL*^*null*^ recipients shows that non-responding (CFSE^high^) CD8^+^ donor cells failed to load PSL2 (Fig 5D(a)) whereas responding (CFSE diluted) donor-derived activated T cells loaded with PSL2 (Fig 5D(b)). Importantly, P-selectin staining was also detected on a subset of recipient CD8^+^ and CD8^neg^ cells (Fig 5D(c/d) and this signal was eliminated by EDTA pre-wash, paralleling the loss of PSL2 on similarly treated donor-derived activated T cells (Fig 5D (b/e)). The phenotype of CD8^+^ cells binding P-selectin in untreated lymph nodes was explored further based on CD44/Ly6c phenotype (Fig 5E); P-selectin binding was significant only on the memory phenotype Ly6C^high^ CD44^high^ subset of CD8^+^ T cells (Fig 5F). Again, display of this P-selectin ligand was *C2GnT1*-dependent and eliminated by EDTA pre-washing.

We conclude that the P-selectin ligand signal on recipient CD8^+^ and/or CD8^neg^ lymph node cells was the only plausible source of PSL2 ligand transferred in Fig 5A; this P-selectin ligand signal was PSGL1-independent, EDTA-elutable, C2GnT1-dependent, and the only other detectable source of P-selectin ligand in the cell suspension available for in vitro loading onto PSL2^negative^ activated T cells. Although likely originating from HEC, the data suggest a ‘pool’ of PSL2 exists on cells in lymph node that can supply PSL2 to T cells undergoing activation. Whether such PSL2 transfer occurs in vivo, as well as its physiological significance, is currently unknown but the transfer was efficient (rapid and significant) and appeared to require only cell:cell contact.

## Discussion

We describe the discovery and analysis of PSL2, a PSGL1-independent P-selectin ligand acquired by responding CD8^+^ T cells after activation in lymph nodes. PSL2 is an unusual selectin ligand in that it is cell-extrinsic, contrasting with PSGL1 and other physiologically active selectin ligands whose expression is cell-intrinsic. PSL2 association with the surface of activated T cells was Ca^++^ dependent, paralleled L-selectin expression, and correlated with staining for L-selectin ligand (Meca79), consistent with PSL2 binding L-selectin via its lectin domain. We conclude that PSL2 is a dual ligand for P- and L-selectin, able to physically bridge both selectins simultaneously. Importantly, PSL2 was the only P-selectin ligand other than PSGL1 detected on primary day-3 activated T cells in the models used.

PSL2 expression levels on activated T cells harvested from lymph node significantly exceeded that detected on activated T cells obtained from spleen. Given that high endothelial venous cells in lymph nodes are a unique source of L-selectin ligands and that these cells are absent in splenic vasculature, we anticipate that PSL2 is originally derived from lymph node HEC.

While the existence of additional selectin ligands has been anticipated [1] PSL2 has not been documented in prior analyses. The cell-extrinsic nature of PSL2, its absence on activated T cells generated in vitro, the rapid rate of its disappearance from activated T cells cultured ex-vivo, and the ease of PSL2 dissociation in media lacking Ca^++^ may account for why PSL2 has not been previously described. Furthermore, much of the effort to identify selectin ligands in the past has been based on leukocytes (neutrophils and myeloid cell lines)[1] or in vitro expanded T cells that will not likely express PSL2. While our study focused on the primary CD8^+^ T cell response in mice, preliminary observations using the *OTII* mice suggested that some EDTA-strippable P-selectin ligand is also present on activated CD4^+^ T cells.

In the assay models used in our study PSL2 was expressed at significant levels on activated T cells, in many cases at levels comparable to PSGL1. We noted however, that the relative expression of PSL2 and PSGL1 expression can vary. It is possible that under different physiological situations either PSGL1 or PSL2 may predominate. When present as a minority of the total P-selectin ligand expressed, either ligand may still contribute signaling input to support recruitment or cell programming for downstream behavior/function.

The identity of PSL2 remains unknown; its cell-extrinsic sourcing, its low abundance, and the low frequency of activated T cells stymied efforts to identify it. As a cell-extrinsic ligand likely sourced from HEC, PSL2 could be either a secreted product or cleaved from a membrane anchored selectin ligand. L-selectin ligands known to be secreted from HEC include Glycam1, Sgp200, and Parm1. As noted above, we re-derived the *Glycam1*^*null*^ mouse but PSL2 detection on activated T cells generated in *Glycam1*^*null*^ recipients was unaltered, ruling it out as a PSL2 candidate. Sgp200 and PARM-1 remain candidates for PSL2. Sgp200 is a minimally characterized 200kD sulfated glycoprotein [18, 29–31]. PARM-1 is synthesized in HEC [32] and weakly secreted [33].

In addition to PSL2 identity, its physiological relevance is also a remaining question. Multiple membrane-anchored ligands have been identified for P-selectin and L-selectin but in many cases, and for P-selectin ligands in particular, their physiological contributions remain ambiguous. Twenty years ago criteria were proposed [34] to distinguish those selectin ligands that *do* bind selectins physiologically from those ligands identified in vitro that *can* bind selectins but lack evident physiological roles.

First, a putative selectin ligand should be expressed at a time and place appropriate for its anticipated role in a selectin-supported process, such as recruitment. Primary immune activation to infection is normally stimulated in secondary lymphoid organs where responding T cells are conditioned for recruitment by induction of selectin ligand. PSL2 is indeed displayed by primary activated T cells responding in lymph nodes.

Second, absence of a putative selectin ligand should be paralleled by loss of physiologically relevant selectin interactions. PSL2 removal reduced P-selectin-dependent adhesion of activated T cells to immobilized P-selectin and to activated platelets.

Third, selectin should show specificity for the ligand and engage it with reasonably high affinity. Selectin binding to PSL2 was Ca^++^ dependent, of sufficient functional affinity to permit both stable labeling of ex-vivo T cells with P-selectin chimera and stable adhesion of cells to P-selectin bearing substrates. We conclude that according to these criteria PSL2 is likely to be physiologically relevant.

What would be the utility of acquiring a cell-extrinsic P-selectin ligand on L-selectin? Adding a second P-selectin ligand to the cell surface could simply enhance physical adhesiveness of activated T cells to substrates bearing P-selectin; indeed data presented here are consistent with PSL2 cooperating with PSGL1 for physical adhesion. However, since activated T cells present PSL2 in complex with cell surface L-selectin, L-selectin too will be indirectly engaged when such substrates are encountered. Given L-selectin’s signaling involvement in both homing and recruitment it is reasonable to suspect that, aside from physical adhesion, additional consequences would accrue from L-selectin being drawn into engagement with P-selectin. Several known aspects of L-selectin distribution and function are salient to this discussion.

First, in several distinct systems L-selectin is limiting for important physiological outcomes including lymphocyte entry into lymph node [22, 23, 35], neutrophil priming [36], neutrophil recruitment in inflammation [37], neutrophil competence for response to chemotactic cues [38–40], and T cell mediated viral clearance [20].

Second, L-selectin is generally thought to exist as a monomer on the cell surface [41, 42]. However, there is evidence that T cell activation results in L-selectin oligomerization and increased functional affinity for its ligands [43–48]. These data are consistent with our observation that only responding donor T cells, but not quiescent donor cells, loaded PSL2 onto L-selectin. Clearly, the enhanced binding capacity of L-selectin for PSL2 on responding cells is secondary to T cell activation and not to L-selectin-stimulated clustering after crosslinking by putative counter-receptor [49] since such a mechanism would be equally available to both quiescent and responding cells in lymph nodes.

Third, PSGL1 and L-selectin are both thought to reside in physical proximity at the tips of microvilli [50]. A functional complex between L-selectin and PSGL1 has been described in mouse [51] and human [52] neutrophils that is reportedly effective for P-selectin-stimulated integrin activation. P-selectin engagement of PSGL1 can stimulate integrin activation [3, 53–56] while L-selectin signaling can enhance responsiveness to chemokines [38–40]. Proximal signaling though PSGL1 and L-selectin might generate mutually reinforcing signals to facilitate adherence and/or post-adherence behavior of activated T cells after encounter with P-selectin. Our observation that PSL2/L-selectin on activated T cells supported adherence to activated platelets via P-Selectin introduces the possibility that such interactions supplement those between PSGL1 and P-selectin, and thought to drive platelet-stimulated recruitment [57–60] and chemokinetic responses [61].

Fourth, it has been reported that O-glycan extensions on selectin ligands such as PSGL1 are required for their sorting to lipid rafts on leukocytes [62]. Although present in both lipid raft domains and non-raft domains [63, 64] PSGL1 signaling was dependent on lipid raft integrity suggesting that only raft-associated PSGL1 molecules are signaling-competent[54, 63]. The presence of L-selectin in lipid rafts is less clear [63, 64] but perhaps ‘dressing’ L-selectin with O-glycans attached to PSL2, L-selectin might be ushered into signaling-competent raft domains and in proximity to PSGL1 on the surface of activated T cells.

**Fig 6:**
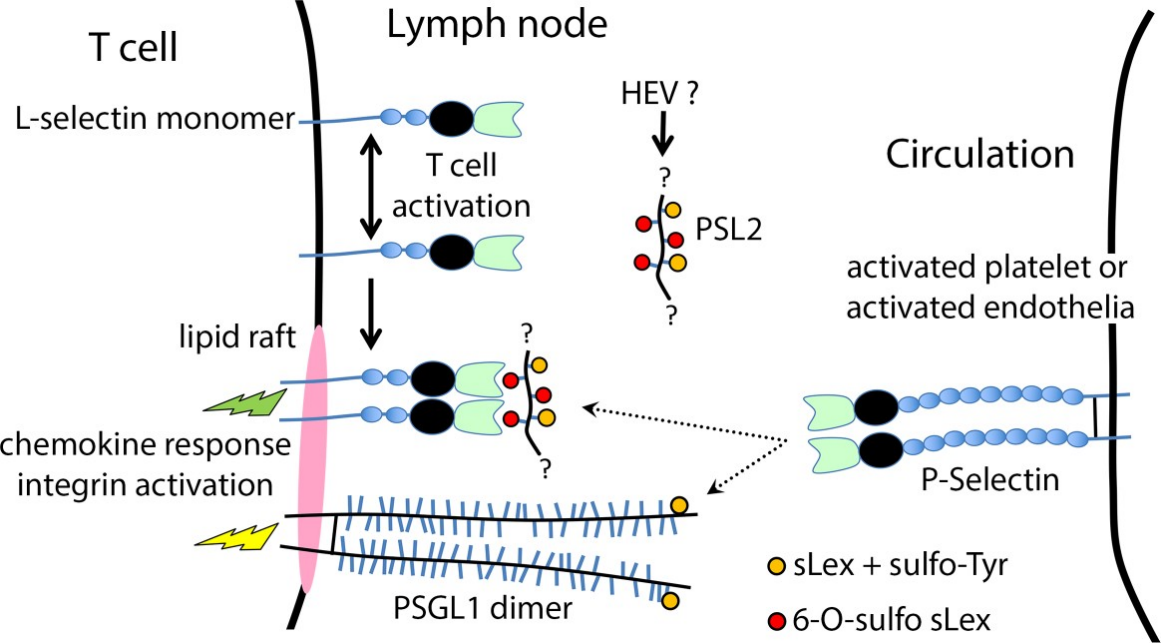
Model of P-selectin ligand expression and activity on primary activated CD8+ T cells consistent with prior literature and data. **PSL2 structure:** PSL2 association with activated T cells requires calcium and is quantitatively dependent on L-selectin. PSL2 is likely an L-selectin ligand produced by lymph node HEV and bearing 6-O-sulfo sLex, the L-selectin-binding structure shared by all known L-selectin ligands. 6-O-sulfo sLex is also likely present on the exposed, unengaged, face of PSL2 since Meca79 staining correlated with PSL2 expression on both recipient and responding-donor CD8^+^ T cells (Figs 3F and 4D); Meca79 recognizes 6-O-sulfo sLex on core 1 extensions. P-selectin recognition of the canonical P-selectin ligand PSGL1 requires both sLex and proximal tyrosine sulfation; whether PSL2 bound to L-selectin on T cells also presents sLex/Tyr-SO_4_ for P-selectin binding or whether the externally exposed 6-O-sulfo sLex detected by Meca79 can substitute as P-selectin ligand is unknown. In either case PSL2 must present (multiple?) selectin ligands on two faces, available for concurrent binding of L-selectin and P-selectin, and most consistent with PSL2 being a mucin-type glycoprotein. It is possible that PSL2 can only serve as a P-selectin ligand after binding/stabilization by L-selectin. **PSL2 loading onto activated T cells.** PSL2 loading onto CD8^+^ T cells occurs most clearly after their activation in lymph nodes consistent with available literature that activation is accompanied by enhanced functional affinity of L-selectin for its ligands, perhaps through L-selectin dimerization. L-selectin on activated cells effectively acquires PSL2 by extraction from either resident lymphocytes (Fig 5) or from the fluid phase after its presumed secretion by HEV. **Contribution of PSL2 to P-selectin driven signaling and adhesion.** Both L-selectin and PSGL1 are thought to co-reside at tips of microvilli. Once bound to L-selectin, sLex glycans on PSL2 may support L-selectin relocation to lipid raft signaling platforms and in proximity to PSGL1. Upon engagement of P-selectin on activated platelets or activated endothelia, the two proximate P-selectin ligands may provide avidity enhancements along with distinct signaling inputs through PSGL1 and L-selectin supporting integrin activation and chemokine responses respectively.

We therefore propose a model (Fig 6) where L-selectin is reconfigured during T cell activation in lymph nodes enhancing its functional affinity for PSL2. The loading of PSL2 onto L-selectin may support its relocation into lipid raft domains in proximity to PSGL1 thereby presenting two proximate ligands for engagement with P-selectin and providing avidity and co-signaling enhancements to promote both integrin activation and chemokine responses. The participation of PSL2 in support of L-selectin signaling needs to be verified but the downstream implications of cooperation between PSGL1 and the L-selectin/PSL2 complex could be significant. In the case of activated T cells where proper chemokine responsiveness is required for CD8 effector T cells locating infected cells in tissue[65, 66], and antigen-encounter secures resident memory CD8^+^ T cell development[67], engagement of L-selectin in the primary response could contribute to the effective recruitment and further development of effector T cells.

The finding that a cell-extrinsic ligand, in this case PSL2, can mediate interaction between two selectins is unprecedented in the context of a large selectin literature. However, observation of direct selectin:selectin connectivity is not without precedent. In contrast to mouse L-selectin, human L-selectin on both neutrophils and activated lymphocytes is modified by O-glycans for recognition by E-selectin[68, 69] enabling direct L-selectin:E-selectin engagement. Importantly, E-selectin in humans is thought to supplant the prominent role played by P-selectin in mice for tethering and recruitment[70]. In contrast to other E-selectin ligands on human neutrophils (eg. PSGL1 and sialylated glycosphingolipids), E-selectin binding to L-selectin can directly trigger the high affinity conformation of LFA-1 integrin, independent of supplementary signals from G-protein coupled receptors [49]. In humans therefore, L-selectin:E-selectin connectivity provides direct and distinctive signaling input relevant to recruitment. The L-selectin:PSL2:P-selectin connectivity we describe for murine T cells may parallel the human L-selectin:E-selectin connectivity, both systems integrating L-selectin engagement upon leukocyte contact with endothelial selectins in inflammation. Analysis of PSL2 function in mice offers an opportunity to isolate L-selectin’s role during recruitment versus homing to lymph nodes and the functional importance of such L-selectin connectivity in vivo.

Although PSL2 is capable of adhesive activity cooperating with PSGL1 for P-selectin-bearing substrates, the contribution of PSL2 to T cell recruitment in vivo needs to be confirmed with development of the PSL2 knockout mouse. It was recently reported in an influenza infection model that levels of L-selectin expression on activated T cells, early in the anti-flu response, correlated with both recruitment of CD8^+^ effector T cells and influenza virus clearance[20]. In a confounding twist, no L-selectin ligand (Meca79 binding) could be detected on vasculature of infected tissue. The discovery of an L-selectin/PSL2 complex on activated T cells offers one possible explanation to reconcile these data whereby PSL2 loading onto L-selectin may enable effector T cell recruitment through PSL2 tethering to endothelial P-selectin; the ‘missing L-selectin ligand’ on inflamed endothelium used by flu specific effector T cells may be P-selectin itself.

## Conclusions

We present the discovery of a new P-selectin ligand, PSL2, acquired by activated T cells responding to antigen stimulation in peripheral lymph nodes. PSL2 is resolved as a cell-extrinsic P-selectin ligand bound to L-selectin on activated T cells. The L-selectin/PSL2 complex can engage P-selectin and co-operate with PSGL1 for adhesion to P-selectin-bearing substrates. The capacity of PSL2 to simultaneously bind and bridge P-selectin and L-selectin adds a new dimension to how, ‘dressed’ as a P-selectin ligand, L-selectin on activated T cells may support recruitment by delivering signaling input driven by engagement with P-selectin on activated endothelia and/or activated platelets. Our results also extend the scope of selectin ligand function by demonstrating how a cell-extrinsic soluble selectin ligand can enhance connectivity between distinct selectins.

## Supporting information

S1 Fig Activated CD8+ T cells responding in *Glycam1^-/-^* recipients load PSL2 normally. (PDF)

S2 Fig PSL2 is recognized by P, E, and L-selectin. (PDF)

S3 Fig PSL2 detection on activated T cells by P-selectin-hIgG is dependent on sialic acid. (PDF)
