# Supplementary material for: A cell-extrinsic ligand acquired by activated T cells in lymph node can bridge L-selectin and P-selectin
Source: PLoS One. 2018 Oct 31;13(10):e0205685. doi: 10.1371/journal.pone.0205685 (PMC6209203; doi:10.1371/journal.pone.0205685)
Supplement: S1 Fig — (PDF) [file pone.0205685.s001.pdf]

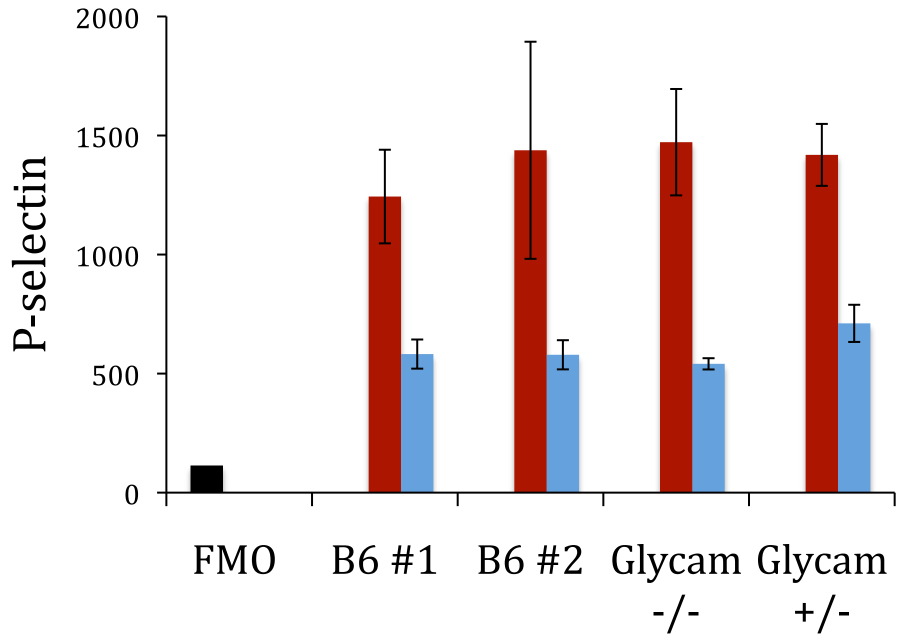

S1 Fig. Activated CD8+ T cells responding in *Glycam1*$^{-/-}$ recipients load PSL2 normally. *OT1-C2GnT1*$^{-/-}$ donor cells were activated for three days in two control B6 recipients, a *Glycam1*$^{-/-}$ recipient, or a *Glycam1*$^{+/-}$ littermate recipient. Peripheral lymph nodes were harvested and either untreated (*red*), or subjected to an EDTA pre-wash to strip PSL2 (*blue*), prior to staining with P-selectin-hIgG followed by anti-hIgG-PE and anti-CD8-APC. Fluorescence-minus-one (FMO) control (*black*) staining of a B6 sample where only P-selectin-hIgG was omitted. Gated analysis of CD8^{+}, PI^{negative}, singlets shown. Figure shown is representative of three independent analyses. Error bars correspond to one standard deviation of triplicate stains of each sample.
