# Supplementary material for: A cell-extrinsic ligand acquired by activated T cells in lymph node can bridge L-selectin and P-selectin
Source: PLoS One. 2018 Oct 31;13(10):e0205685. doi: 10.1371/journal.pone.0205685 (PMC6209203; doi:10.1371/journal.pone.0205685)
Supplement: S2 Fig — (PDF) [file pone.0205685.s002.pdf]

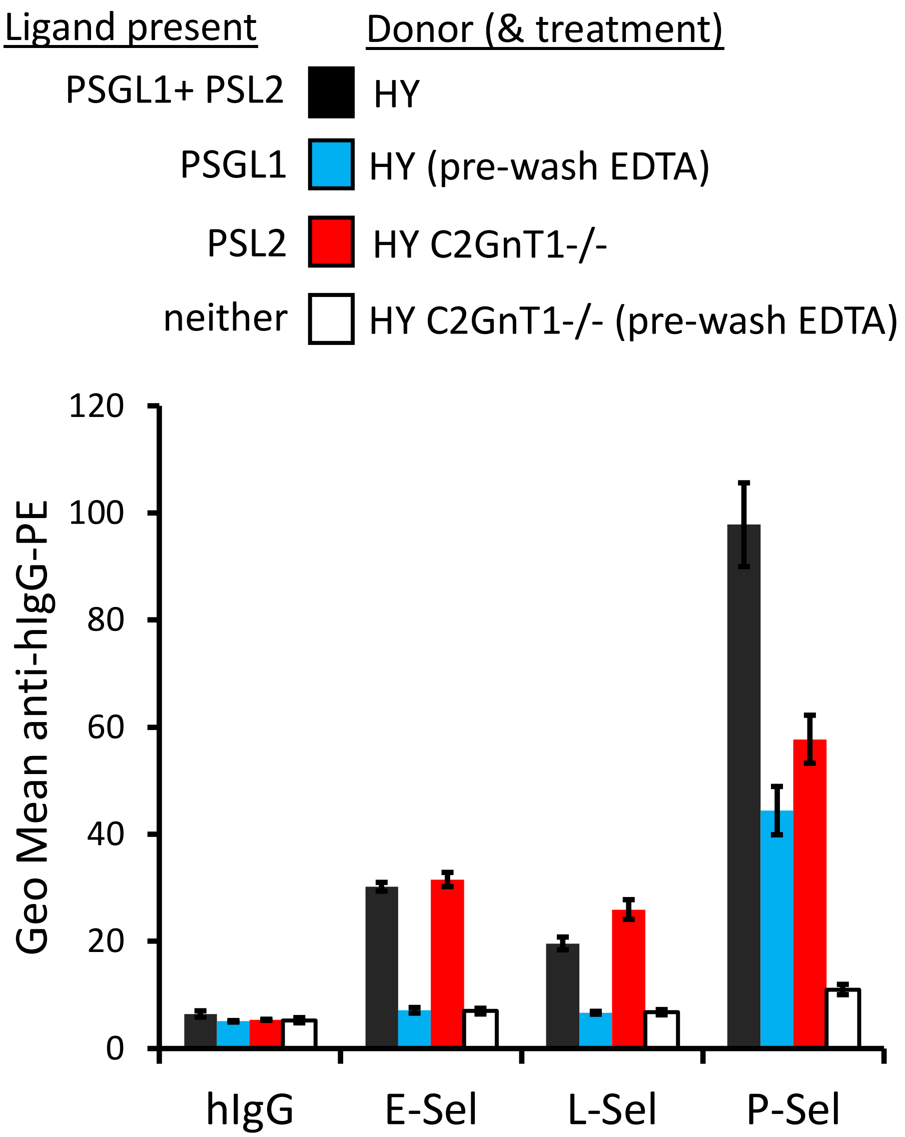

S2 Fig. **PSL2 is recognized by P, E, and L-selectin**. CFSE-labeled donor *HY* or *HYC2GnT1*^{null} cells were transferred into *PSGL1*^{null} *Thy1.1* male recipients and recovered from peripheral lymph nodes on day 3. Aliquots of these cells were either left untreated or EDTA pre-washed and returned to Ca^{2+} replete media for staining. In this way, four donor-derived populations with distinct profiles of PSGL1 and PSL2 could be compared for binding of E-, L-, or P-selectin. Cells were stained with 5 µg/ml of hIgG1 chimeras of each of the murine selectin followed by anti-hIgG-PE, CD8-APC and propidium iodide.

PSL2 was bound to some degree by all selectins and binding eliminated by EDTA pre-washing. Geometric mean fluorescence with standard deviation of three staining replicates of each population indicated. Samples were gated on responding (CFSE-diluted), $CD8^+$, propidium iodide-negative events. Results were consistent among five independent experiments.
