# Supplementary material for: A cell-extrinsic ligand acquired by activated T cells in lymph node can bridge L-selectin and P-selectin
Source: PLoS One. 2018 Oct 31;13(10):e0205685. doi: 10.1371/journal.pone.0205685 (PMC6209203; doi:10.1371/journal.pone.0205685)
Supplement: S3 Fig — (PDF) [file pone.0205685.s003.pdf]

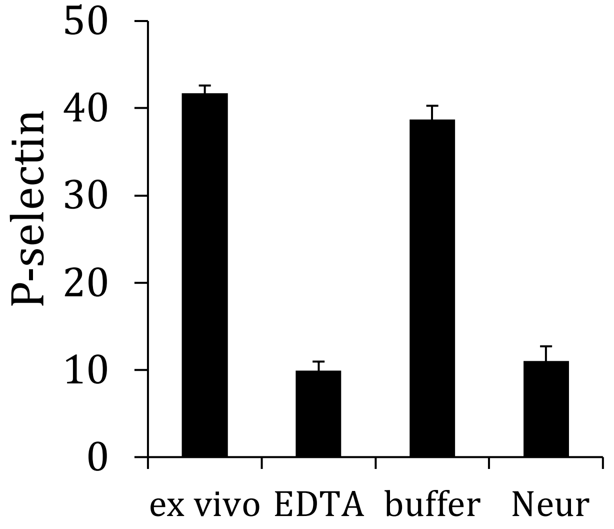

S3 Fig. PSL2 detection on activated T cells by P-selectin-hIgG is dependent on sialic acid. *HY-C2GnT1^{null}* donor cells responding in lymph nodes of male *PSGL1^{null}* recipients were harvested and stained on day 3. P-selectin staining of PSL2 was prevented either by EDTA pre-wash (*EDTA*) or by exposure to neuraminidase (*Neur*) but not enzyme reaction buffer alone (*buffer*). Cells, 10^6 cells per sample, were re-suspended in H^+ pre-acidified with HCl to pH 5.8 and supplemented with 2.5% fetal calf serum. Neuraminidase Clostridium Perfringens (Roche #11 585 886 001 stock at 25 U/ml) was added to 125 mU/ml final concentration and the mixture incubated at room temperature for 15 minutes. Fetal calf serum was then added to a final concentration of 30% to reduce cell aggregation, and samples placed on ice for 5 minutes. They were then washed in I_{10} and stained with P-selectin chimera. Geometric mean fluorescence and standard deviation shown. Figure shown is representative of three independent analyses.
